# The Relationship Between Observers' Self-Attractiveness and Preference for Physical Dimorphism: A Meta-Analysis

**DOI:** 10.3389/fpsyg.2018.02431

**Published:** 2018-12-05

**Authors:** Lijun Chen, Xiaoliu Jiang, Huiyong Fan, Ying Yang, Zhihong Ren

**Affiliations:** ^1^School of Humanities and Social Sciences, Fuzhou University, Fuzhou, China; ^2^Institute of Psychological and Cognitive Sciences, Fuzhou University, Fuzhou, China; ^3^College of Teacher, Bohai University, Jinzhou, China; ^4^Key Laboratory for Adolescent Cyberpsychology and Behavior of the Education Ministry, Wuhan, China; ^5^Laboratory of Human Development and Mental Health, Institute of Psychology, Central China Normal University, Wuhan, China

**Keywords:** femininity, masculinity, meta-analysis, self-attractiveness, sexual dimorphism

## Abstract

**Background:** Many studies have reported an association between observers' self-attractiveness and their preference for sexual dimorphism across different physical domains, including the face, voice, and body. However, the results of these studies are inconsistent. Here, a meta-analysis was conducted to estimate the association between observers' own attractiveness and their dimorphic preference.

**Methods:** Major electronic databases including PsycINFO, Web of Science, PubMed, ProQuest, and Google Scholar were searched during April 2017 (the first time) and April 2018 (the second time). The effect size computation and moderating effect analyses were conducted separately for masculine and feminine preferences.

**Results:** We identified 5,359 references, of which we included 25 studies (*x* = 55, *x* = number of the effect size) with 6,853 participants in the meta-analysis. Across these studies, the correlation between observers' own attractiveness and their sexual dimorphic preference was 0.095 (*x* = 55) and that for preference for masculinity (*x* = 39) and femininity (*x* = 16) were 0.102 and 0.076, respectively. The results of the funnel plot, Egger's regression method, and fail-safe number suggested that there was no obvious publication bias. The relationship depended on the relationship context (short or long-term), opposite or same sex (the gender of the observer and host), measures of observers' self-attractiveness (subject or objective), and preference task (e.g., attractiveness rating, forced-choice, and face sequence test). Furthermore, for female participants, using a hormonal contraceptive also influenced their masculinity preference. The effect size for the preference for a masculine body and voice was larger than that for facial masculinity.

**Conclusion:** We found a small but significant correlation between self-attractiveness and physical dimorphic preference, the relationship was moderated by the relationship context, same/opposite-sex, and contraceptive using. These three moderating effects represented the observer's trade-off on good genes, good provider and good father (3Gs) consistent with the life history strategies. Besides, measurement of observers' attractiveness, type of preference task and stimuli may also involve the relationship.

## Introduction

Secondary sexual characteristics in adult humans reflect the masculinization or feminization that occurs during puberty (Perrett et al., 1998; Rhodes, 2006). Physical sexual dimorphism is a broad concept that could include sexual dimorphism in multiple domains (e.g., face, body, voice). Sexually dimorphic physical traits are important for mate choice and mate preference in many species, including humans. Several previous studies have observed that humans' preferences for physical cues of extreme secondary sexual characteristics (more feminine for women, more masculine for men) in different domains (e.g., visual, vocal, and bodily) are correlated (Little et al., 2007; Fraccaro et al., 2010). These correlations demonstrate a systematic, rather than arbitrary, variation in humans' preferences for sexual dimorphism, which are consistent with the proposal that sexually dimorphic cues in different domains reflect a common underlying aspect of quality. On the evolutionary view, femininity of women, masculinity of men are proposed to be more attractive because they advertise the good genes of an individual (Rhodes, 2006). Among humans, physical characteristics consistent with the owner's gender are correlated with indices of long-term health (Rhodes et al., 2003; Thornhill and Gangestad, 2006), reproductive potential (Puts, 2005; Rhodes et al., 2005), and low parasite loadings and high immune competence (Thornhill and Gangestad, 1993, 1996), but negatively correlated with prosociality (Haselton, 2005; Haselton and Gangestad, 2006). Men's masculine traits indicate untrustworthiness and bad parental traits (Boothroyd et al., 2007; Smith et al., 2009b), and women's femininity are considered as more likely to be unfaithful, to pursue short-term relationships, and to be in higher risk of cuckoldry (Little et al., 2014).

According to the life history (LH, referring to organisms capturing energy from the environment and using it to produce more organisms) trade-off model strategies in mating choice (Gangestad and Simpson, 2000; Del Giudice and Belsky, 2011), women focused on two types of characteristics when they chose a mate: those indicating a “good provider” (social-economic characteristics, such as wealth, education, career) and “good genes”(physical characteristics) (Gangestad and Buss, 1993; Gangestad and Simpson, 2000). Other researchers believed that the framework of women's mate preference should involve three Gs. Besides good genes, good providers, women also prioritize a man's personality traits—for example, being kind, loving, and staying at home—that constitute good fathers (Buss and Shackelford, 2008; Lu et al., 2015). Good provider indicate men have resource to invest in parenting, traits of good father can reflect men's intention to help raising young, both of the two types of characteristics represent parenting of reproductive effort in post mating events (parental investment), otherwise good genes characteristics were realized as mating attributes affecting premating decisions. When exposed to contrasting environments, women would have evolved to make trade-offs between investment qualities and indicators of good genes contingent on specific environmental conditions, because good genetic males tend to have more mates at the same time, and they invest in each female than in males of lower phenotypic quality, women's emphasis on good genes may be at the cost of men's parental investment (Gangestad and Simpson, 2000). Men also encounter trade-off problems, because, while feminine females possess high attractiveness and good genes, they are also associated with negative personality characteristics (unfaithful) (Haselton, 2005; Haselton and Gangestad, 2006), and men also have expectations about maternal investment. For both men and women (but more for men than women), parental warmth and care (good father or mother) correlated negatively with good-gene and good-provider mate values (Chang et al., 2017). Both men and women encounter the tradeoff of 3Gs mating framework.

Therefore, preferences for dimorphism represent the result of trading off between good genes and parental investment. Differences in how humans resolve this trade-off can lead to individual differences in sexual dimorphic preference. For example, attractive women demonstrate stronger preferences for masculine men than relatively unattractive women do (Little et al., 2001; Little and Mannion, 2006). These are called condition-dependent preferences. In the evolution of species, condition-dependent preferences have been observed in many species, in which individuals in good physical condition tend to show stronger preferences for high-quality mates (Bakker et al., 1999). Condition-dependent preferences in both humans and non-humans may have a common function and they may occur because individuals in good physical condition (i.e., attractive individuals) are better able to compete for and/or retain high-quality mates (Little et al., 2001). Additionally, they can offset the costs of choosing a partner with good genes (e.g., by being able to replace a partner more quickly), and therefore, they improve their criteria for mate selection. On the contrary, in order to meet the needs of parental investment, individuals with poor self-conditions may reduce their standards for mate selection, and they prefer mates who are more likely to make high parental investment. The following findings appear to be somewhat analogous to condition-dependent preferences observed in humans. Women's ratings of their own physical attractiveness positively correlated with the strength of their preferences for masculine characteristics in men's faces (Little et al., 2001; Penton-Voak et al., 2003; Smith et al., 2009b). Similar correlations have been found in men's voices (Vukovic et al., 2008, 2010) and bodies (Little et al., 2007). Further, the concept of condition-dependent preferences is conceptualized as “market value dependent preferences”: when exposure to attractive same-sex images, women perceived themselves less attractiveness and lower prefer for male facial masculinity; whereas exposure to unattractive same-sex images, they perceived themselves more attractiveness and lower prefer for masculinity (Little and Mannion, 2006).

There is inconsistent evidence on the relationship between observers' own attractiveness and their sexual dimorphic preference. While some experimental results have confirmed that attractive females prefer masculine faces (Smith et al., 2009b; Welling et al., 2009; Kandrik and DeBruine, 2012), others did not find such a relationship (Zietsch et al., 2015; Carrito et al., 2016). However, several studies have found that observers' own attractiveness can interact with other variables and impact their preferences (Little et al., 2001; Smith et al., 2009a; Burriss et al., 2011; Chen et al., 2017).

### Influence of Relationship Context in the Preference of Opposite-Sex

The context in which judgments are made can also contribute to differences in the relationship between dimorphic preference and the observer's self-attractiveness (Little et al., 2001, 2002; Penton-Voak et al., 2003; Carrito et al., 2016). There are two types of relationship context: short-term and long-term. The former refers to a sexual relationship, such as a one-night stand; the latter is a lasting relationship, such as being married. The trade-off theory proposed that contextual factors affect the strength of people's preferences for a masculine or feminine partner (Gangestad and Simpson, 2000). Attractive women prefer more masculine male faces than less attractive women do, and this difference is seen in the context of a long- but not a short-term relationship (Penton-Voak et al., 2003). This result has been supported by several studies across voice and body stimuli (Little et al., 2007; Feinberg et al., 2012). Otherwise, attractive men exhibit stronger preferences for feminine women only in the short-term context (Burriss et al., 2011; Little et al., 2014). As feminine women were seen as more likely to be unfaithful and more likely to pursue short-term relationships (Boothroyd et al., 2008), and the risk of cuckoldry limit men's preferences for femininity in women and that it could additionally lead to preferences for femininity in short-term mates (Little et al., 2014). Thus, we supposed that the influence of attractiveness on male masculine preferences is more pronounced in the long-term than in the short-term context for women, but for men, the effect of attractiveness on female feminine preference is more prominent in the short-term context.

### Observer's Age and Contraceptive Using

It has been found that reproductively active women had the strongest preference for males' masculinity (Little et al., 2010; Jones et al., 2011), and another study only observed the age effect in women using no contraception (Little et al., 2002). Some studies have found that hormonal contraceptive use may modulate individual differences in women's masculinity preference (Little et al., 2002; Feinberg et al., 2008; Smith et al., 2009a). Furthermore, Vukovic et al. (2008) found that self-rated attractiveness was positively related to the strength of women's preference for masculinized men's voices in women reporting no use of hormonal contraceptives, but not in those using the same. Actually, effects of female observer's age and contraceptive using both due to their physiological hormone levels. Because females of different ages are in different reproductive stages with different hormone levels. And women using hormonal contraceptives are in a hormonal state similar to pregnancy, and consequently, they are unable to realize the benefits that are thought to be associated with choosing a masculine mate (i.e., increased offspring health) (Smith et al., 2009a). In light of the previous studies, we speculate that attractive-contingent preference is stronger in women who do not use hormonal contraceptives than in those who do.

### Measures of Observer's Own Attractiveness

Initially, researchers used self-rated items to determine observers' own attractiveness. Little et al. (2001) used self-reported attractiveness as an indicator of observers' own attractiveness and found that attractive females preferred masculine male faces; Little and Mannion (2006) found that women's subjective impressions of their own market value (i.e., their self-rated attractiveness) is particularly important with reference to the effects of attractiveness on women's masculinity preferences. However, other researchers failed to confirm these findings (Cornwell et al., 2006). Therefore, some researchers have questioned the veracity of subjective assessments, and began to adopt objective measures of self-attractiveness in experiments (Penton-Voak et al., 2003). These objective measures included other-rated facial attractiveness, waist-to-hip ratio (WHR), and body mass index (BMI) (Penton-Voak et al., 2003; Smith et al., 2009b). found that women with high WHR (an indicator of unattractiveness) and/or relatively low other-rated facial attractiveness preferred more feminine male faces when choosing males for a long-term relationship. This result has been confirmed by another study (O'Connor et al., 2012). Then, what kind of measurement (subjective or objective) would be more sensitive to self-attractiveness? In the current study, we examined the moderating role of the measurement of observers' own attractiveness (subjective/objective) and compared the effect sizes of the measurements in this relationship.

Thus, evidence on the relationship between observers' self-attractiveness and preference for sexual dimorphism is equivocal. This is potentially because the preference is condition-dependent. Therefore, we can conclude that observers' self-attractiveness is an important variable that interacts with other variables such as measuring methods, relationship context, same/opposite-sex, and contraceptive use (yes or no) to influence observers' dimorphic preferences. Based on the LH trade-off model strategies and condition-dependent preference, the meta-analysis technique was used in the present meta-analysis to investigate whether observers' sexual dimorphic preferences vary across their self-attractiveness, and to interpret the possible reasons for the divergence. We focused on the following two core issues: What is the totally coefficient of the relationship between the two variables? Which factors moderate their relationship significantly?

## Methods

### Information Sources and Search

The following search terms were used in combination: sexual dimorphism, masculin^*^, feminin^*^, fac^*^, bod^*^, vocal, voice, and *attractiveness*. Search terms for observers' own attractiveness included *self-rated attractiveness, self-perceived attractiveness, self-reported attractiveness, self-perceptions of attractiveness, self-ratings of attractiveness, other-rated attractiveness*, and *third-party attractiveness ratings*. Major electronic databases, including PsycINFO, Web of Science, PubMed, ProQuest, and Google Scholar were searched during April 2017(the first time) and April 2018 (the second time). The reference lists of the included studies were searched to identify additional studies.

### Eligibility Criteria and Study Selection

**Figure 1:**
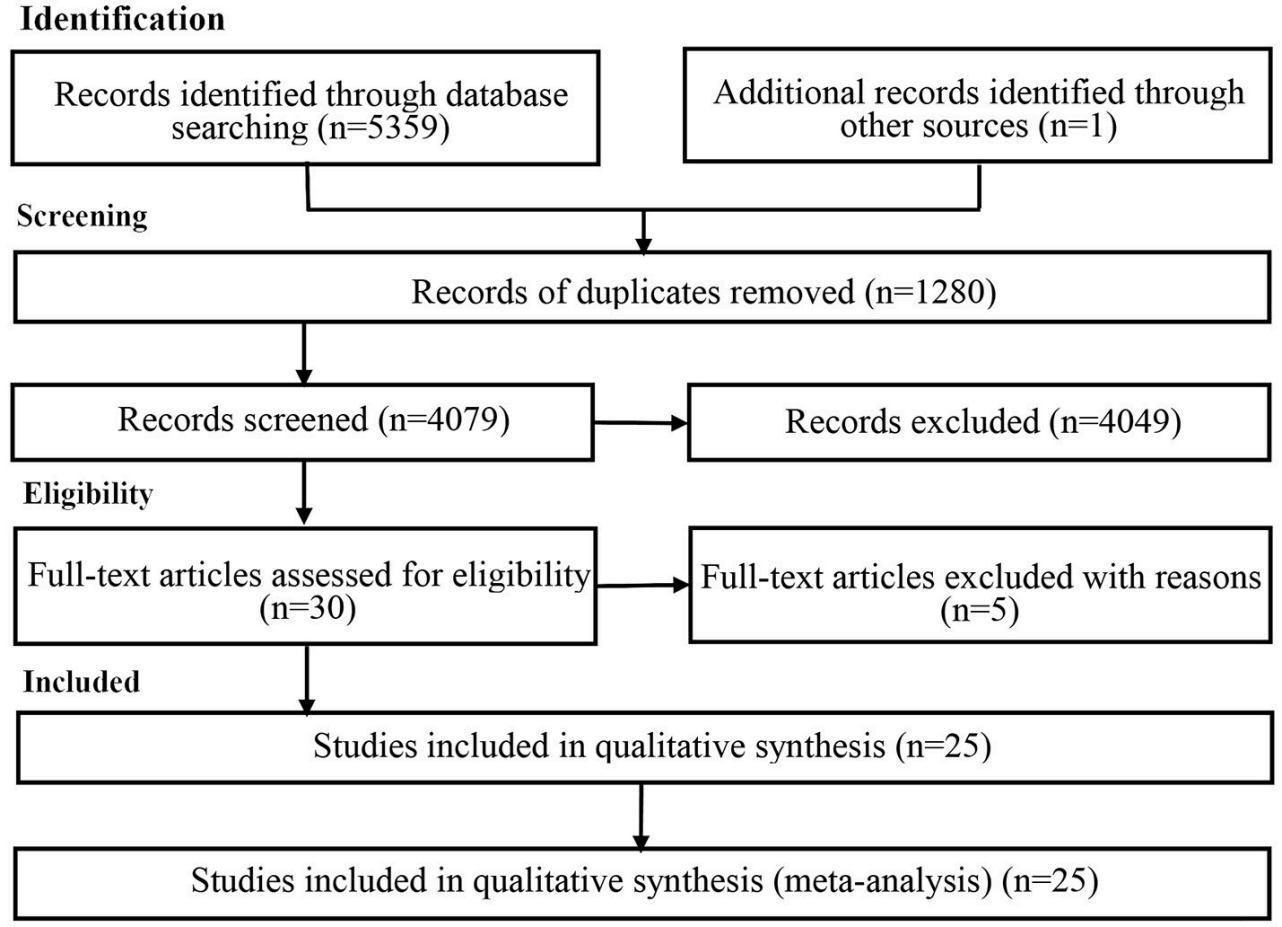
Flow diagram depicting the search protocol and workflow in determining the studies for inclusion in the meta-analysis.

Only studies that met the following three criteria were included: The relationship between the sexual dimorphic preference and observer's self-attractiveness was investigated in the study. Specific data on this relationship were accurately reported in the study (such as the correlation coefficients *r*; mean; standard deviation; sample size; or corresponding *F, t*, or χ^2^*)* to enable the calculation of the effect size, excluding the data of the structural equation model, path analysis, and multivariate regression analysis. In order to avoid missing important literature, we wrote to the authors (first or corresponding author) to obtain the correlation coefficient if it was not reported in the article. In cases where there were multiple reports of the same study, we used the first published report. Two authors independently screened the titles and abstracts of the identified articles to exclude ineligible studies. Disagreements were resolved by discussion. We retrieved the full text of the potentially eligible studies and examined full-text reports for further evaluation. The PRISMA flow diagram (Moher et al., 2010) represents all the steps of the literature search (see Figure 1).

### Summary Measures

In psychological research, the standard mean difference (*d*) and correlation coefficient (*r*) are frequently used to compute effect sizes. In order to integrate the relationship between the sexual dimorphic preference and observers' own attractiveness, the correlation coefficient (*r*) was used in the present meta-analysis. In some primary studies correlation coefficients can be retrieved from *t, F*, or χ^2^ which reported. The following formulas were used in this context (Card, 2012):

$$\begin{array}{r} r=\sqrt{\frac{t^{2}}{t^{2}+df}},df=n_{1}+n_{2}-2;r=\sqrt{\frac{F\left(1,-\right)}{F\left(1,-\right)+df\left(error\right)}};\\ r=\sqrt{\frac{\chi^{2}}{\chi^{2}+N}}. \end{array}$$

After extracted, all correlations were transformed using Fisher's *Z*-transformation (Lipsey and Wilson, 2001). The sample distribution of *Z*_*r*_ is approximately equal to the normal distribution (Hittner and Swickert, 2006; Borenstein et al., 2009). The formula for the transformation is as follows: $Z_{r}=0.5\times \text{ln}\left(\frac{1+r}{1-r}\right)$. The overall *Zr* can be computed through weighted *Zr*. Then the overall *r* can be found through an inverse operation of Fisher' *Z*-transformation (Lipsey and Wilson, 2001; Borenstein et al., 2009). These computation related to overall effect size estimation were conducted under the random-effects model (Borenstein et al., 2009). The specialized statistical software Comprehensive Meta-Analysis (CMA, Version 2.2) was used in the current meta-analysis for conducting all the needed computations and analyses.

### Data Extraction

Two authors extracted the following information: first author's name, year of publication, sample size of observers, observers' age, gender of the stimuli (male or female), observers' gender (male or female), facial attractiveness task (the forced-choice test with an attractiveness rating, the forced-choice test alone, or the face sequence test), sexual dimorphic preference (masculinity or femininity), measures of observers' own attractiveness (objective or subjective), type of stimuli (face, voice, body), and use of contraceptive (yes or no; for female observers only).

Considering that the sexual dimorphic preference is divided into masculine preference and feminine preference, and that the correlation between observers' own attractiveness and their masculine or feminine preferences is opposite, this meta-analysis calculated the effect sizes and conducted moderating effect analyses for masculine and feminine preferences separately. The data is available in Supplementary Table 1.

### Heterogeneity Test

The heterogeneity test was conducted to test whether the average effect size was heterogeneous. Each effect size of each observation value in this meta-analysis contained real and residual effect sizes, which resulted in the partial false phenomenon of effect sizes. The heterogeneity test of effect sizes is always examined by calculating the *Q* statistic (Borenstein et al., 2009) and *I*^2^ (Card, 2012). In this systemic review, the two statistical values of *I*^2^ and *Q* were used to detect the heterogeneity of the included effect sizes.

### Publication Bias

Publication bias is a concern for any meta-analytic review because it can lead to a larger combined effect than what actually exists. This type of bias refers to the phenomenon where published studies are more likely to report larger effects. Because studies that have not been published due to their negative or null findings are more difficult to retrieve, and therefore, are less likely to be included in a meta-analysis, an upward bias in the combined effect may occur. Furthermore, English-language publication are more likely to be searched, which leads to an oversampling of statistically significant studies. In this meta-analysis, we use a variety of methods to minimize and test publication bias. When searching the literature, we also searched the most popular and diverse Chinese database CNKI (China National Knowledge Infrastructure), the conference and the dissertation database; and wrote to the important researchers in this field to ask if they had any unpublished research reports. When analyzing the data, we used the funnel plot, Egger's regression and Rosenthal's Fail-safe *N* to evaluate the publication bias and the degree of its impact (Borenstein et al., 2009).

## Results

### Description of Studies and Overall Association

Due to the topic involved self-attractiveness and dimorphic preference two variables, we combined 7 terms describing preference (face, voice and body) and 6 terms describing observer's attractiveness to search, our literature searches initially identified 5,359 potential articles from databases, but most of them were unrelated articles; Additionally, in many related studies, they involved the relationship between dimorphic preference and self-attractiveness, whereas, they did not look the relationship as an important topic and not describe in the titles, abstracts, keywords, we also searched the references and citing articles of the related studies, which resulted in many duplicates. Finally the current meta-analysis included 25 studies with 6,853 participants (see Table 1). The flow chart has been presented in Figure 1. The 25 eligible studies produced 55 effect sizes because 12 studies consisted of multiple datasets. We examined the relationship between preference for sexual dimorphism and observers' own attractiveness. The results showed that the correlation coefficient *r* (*x* = 55) of the relationship between these two variables was 0.095 (95% *CI*: 0.059, 0.130; *Z* = 5.173, *p* < 0.001). The correlation coefficient *r* of the relationship between observers' own attractiveness and preference for masculinity (*x* = 39) and femininity (*x* = 16) were 0.103 (95% *CI*: 0.060, 0.146; *Z* = 4.691, *p* < 0.001) and 0.076 (95 % *CI:* 0.007, 0.145, *Z* = 2.162, *p* = 0.031 < 0.05), respectively. According to Lipsey and Wilson (2001), an effect size *r* lower than 0.10 indicates a weak correlation. In order to check the stability of the results of the mean effect size analyses, we conducted a sensitivity analysis, which showed that this meta-analysis did not need to eliminate any of the data that had been included. The data is available in Supplementary Table 2.

**Table 1:** Characteristics of the 25 studies included in the present meta-analysis.

**References**	**Task of preference**	**Sexual dimorphism preference**	**Measures of observer's own attractiveness**	**Sample size of observers (*n*)**	**Observer's age**	**Observer's gender**	**Impact factor**
Burriss et al., 2011	Attractiveness rating	Face masculinity	Subjective: self-rated attractiveness	111	20.73 ± 3.37	Men	1.877
	Attractiveness rating	Face masculinity	Objective: other-rated attractiveness	68	–	Men	
Carrito et al., 2016	Face sequence test	Face masculinity	Subjective: self-rated attractiveness	48	22.65 ± 6.60	Women	3.223
	Face sequence test	Face masculinity	Subjective: self-rated attractiveness	61	20.11 ± 4.26	Women	
	Face sequence test	Face masculinity	Subjective: self-rated attractiveness	26	20.39 ± 2.95	Women	
Cornwell et al., 2006	Face sequence test	Face masculinity	Subjective: self-rated attractiveness	46	19.5 ± 1.36	Women	4.997
Feinberg et al., 2012	Sequence test	Voice masculinity	Subjective: self-rated attractiveness	43	–	Women	2.752
Fraccaro et al., 2010	Forced-choice test	Face femininity	Subjective: self-rated attractiveness	178	27.61 ± 9.81	Men	1.278
	Forced-choice test	Voice femininity	Subjective: self-rated attractiveness	178	27.61 ± 9.81	Men	
Holzleitner and Perrett, 2017	Attractiveness rating	Face masculinity	Subjective: self-rated attractiveness	156	18–45	Women	-
Jones et al., 2007	Forced-choice test	Face femininity and masculinity	Subjective: self-rated attractiveness	140	19.84 ± 2.43	Men	2.529
Jones et al., 2010	Forced-choice test	Face masculinity	Subjective: self-rated attractiveness	104	23.3 ± 4.90	Women	1.82
Jones et al., 2011	Forced-choice test	Face femininity	Subjective: self-rated attractiveness	155	48.6 ± 8.1	Women	3.348
Kandrik and DeBruine, 2012	Forced-choice test	Face masculinity	Subjective: self-rated attractiveness	1000	19.5 ± 1.79	Women	1.704
Lefevre and Saxton, 2017	Forced-choice test	Face masculinity	Subjective: self-rated attractiveness	125	20.57 (18–29)	Women	3.223
Little et al., 2001	Face sequence test	Face masculinity	Subjective: self-rated attractiveness	66	22 ± 5.20	Women	3.192
	Face sequence test	Face masculinity	Subjective: self-rated attractiveness	115	22.4 ± 5.4	Women
Little and Mannion, 2006	Forced-choice test and attractiveness rating	Face masculinity	Subjective: self-rated attractiveness	65	23.5 ± 5.6	Women	2.711
	Forced-choice test and attractiveness rating	Face masculinity	Subjective: self-rated attractiveness	75	21.4 ± 3.4	Men	2.254
Little et al., 2014	Face sequence test	Face femininity	Subjective: self-rated attractiveness	393	27.6 ± 6.5	Men	
Little et al., 2007	Forced-choice test	Body masculinity	Subjective: self-rated attractiveness	97	24.9 ± 5.5	Women	3.401
Moore et al., 2011	Face sequence test	Face femininity	Subjective: self-rated attractiveness	212	23.51 ± 9.54	Women men	1.07
O'Connor et al., 2012	Attractiveness rating	Face masculinity	Objective: other-rated attractiveness	63	18.71 ± 1.71	Women	1.947
Penton-Voak et al., 2003	Face sequence test	Face masculinity	Subjective: self-rated attractiveness Objective: other-rated attractiveness, WHR	82	20.2	Women	1.28
Smith et al., 2009b	Forced-choice test and attractiveness rating	Face femininity	Subjective: self-rated attractiveness	147	19.92 ± 3.55	Women	1.278
Smith et al., 2009a	Forced-choice test	Face masculinity	Objective: WHR and BMI	32	–	Women	1.878
Vukovic et al., 2008	Forced-choice test and	Voice masculinity	Subjective: self-rated attractiveness	58+65	19.72 ± 2.85	Women	1.598
Vukovic et al., 2010	Attractiveness rating	Voice masculinity	Objective:	54+58	19.92 ± 2.36	Women	2.926
Welling et al., 2008	Forced-choice test and attractiveness rating	Face femininity	Subjective: self-rated attractiveness	43	19.01 ± 1.52	Women	1.598
Welling et al., 2009	Forced-choice test	Face masculinity	Subjective: self-rated attractiveness	808	18.22 ± 1.09	Women	1.878
Zietsch et al., 2015	Forced-choice test	Face masculinity	Subjective: self-rated attractiveness	2160	33.11 ± 5.00	Women	5.476

### Heterogeneity Test

The overall heterogeneity test (*x* = 55) showed *Q* = 145.567 (*p* < 0.001), *I*^2^ = 62.904, that mean there existed moderate heterogeneity (Higgins et al., 2003). For masculine preferences, the result of the heterogeneity test (*x* = 39) showed *Q* = 72.93 (*p* < 0.001), *I*^2^ = 47.90 (moderate heterogeneity). For feminine preferences, the result of the heterogeneity test (*x* = *16*) showed *Q* = 71.77 (*p* < 0.001), *I*^2^ = 79.10 (high heterogeneity).

Considering that previous studies have shown heterogeneity of sexual dimorphic preferences (Wood et al., 2014), and according to Borenstein et al. (2009), if the true effect varies across studies using different samples, it is more reasonable to use the random model. A large number of studies have shown that sexual dimorphic preferences are influenced by observers' own attractiveness, age, gender, and sexual orientation (Zheng and Zheng, 2016). Therefore, the random model was more suitable for the present meta-analysis.

### Publication Bias

**Figure 2:**
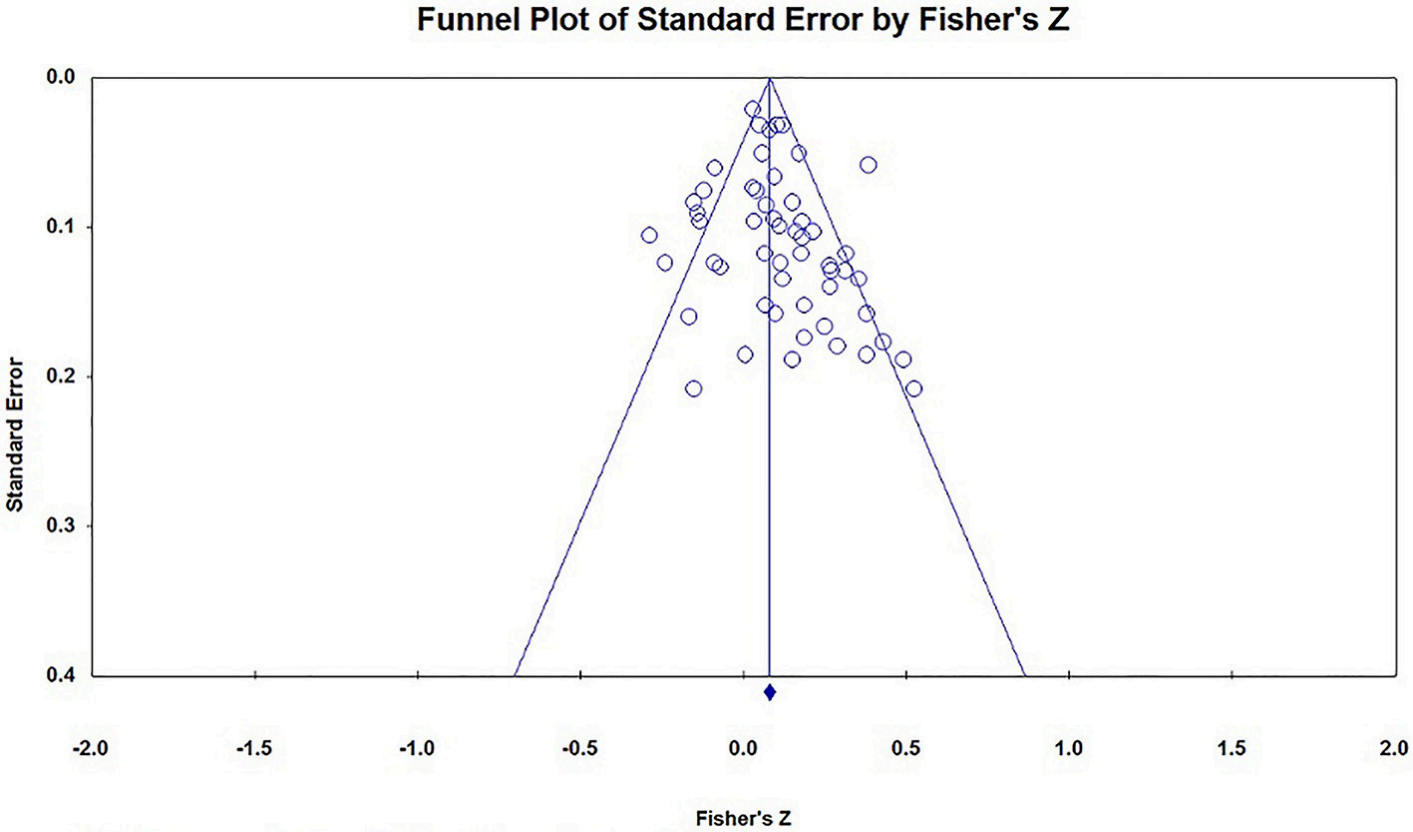
Funnel plot of all the effect size (*x* = 55).

**Figure 3:**
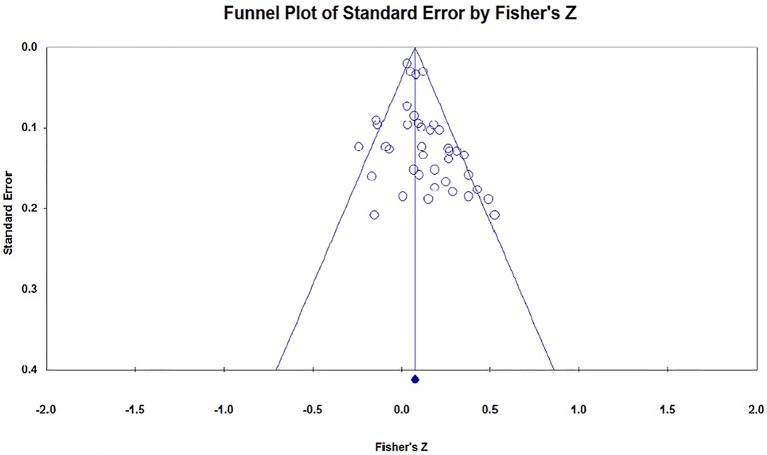
Funnel plot of the effect size of masculine preference (*x* = 39).

**Figure 4:**
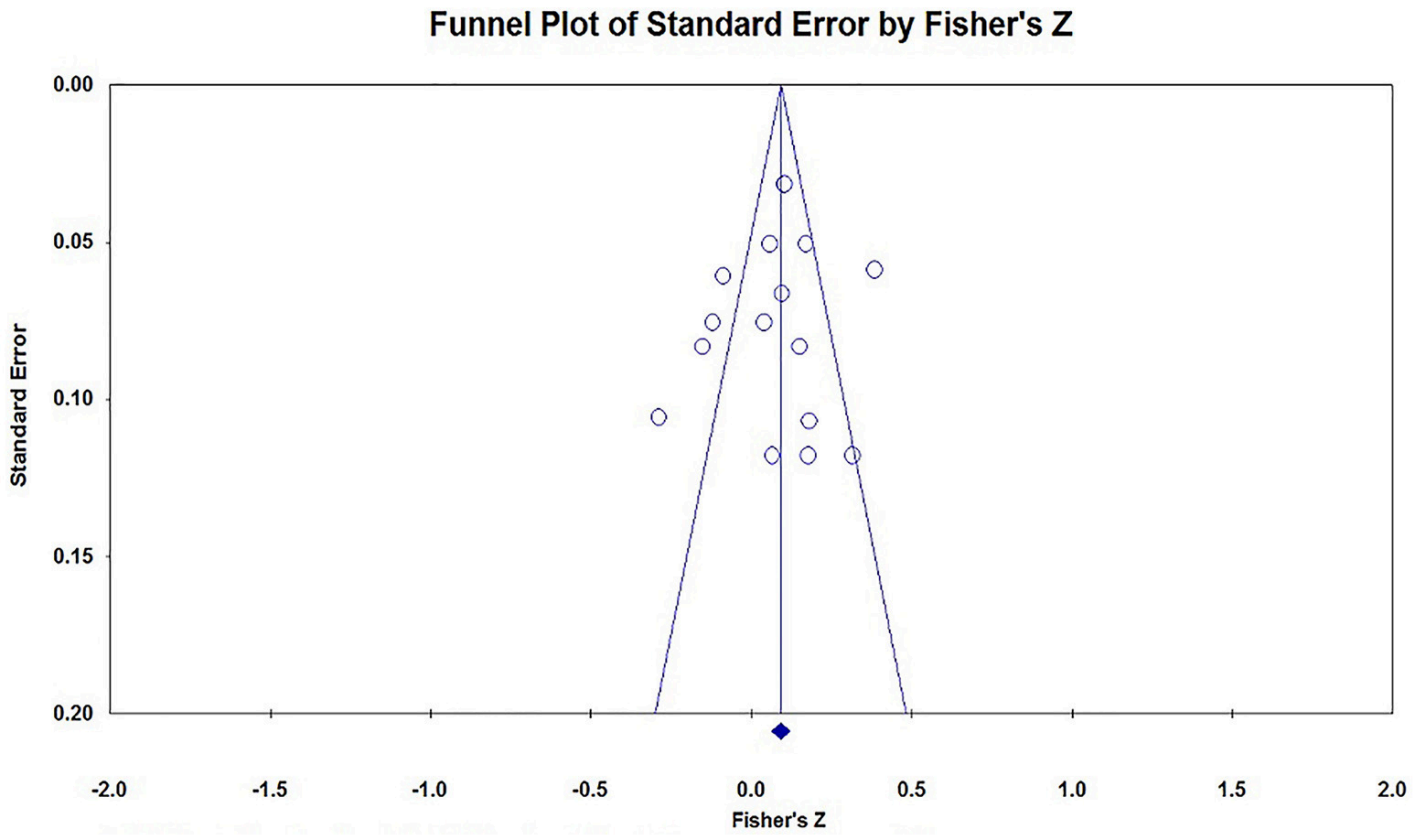
Funnel plot of the effect size feminine preference (*x* = 16).

We could not find any Chinese published researches in the CNKI database, and none of the important researchers said they have unpublished researches on this topic. We used the funnel plot, Egger's regression and Rosenthal's Fail-safe *N* to evaluate the publication bias of the studies included in this meta-analysis. In the absence of publication bias, the studies will be distributed symmetrically about the mean effect size, since the sampling error is random. Otherwise, if the funnel plot is asymmetrical at the bottom, there may be publication bias. The funnel plots in the current analysis are a little asymmetrical at the bottom (see Figures 2–4). Because the interpretation of a funnel plot is largely subjective, the Egger's method has been proposed to quantify or test the publication bias (Egger et al., 1997). So, Egger's method was conducted to detect the publication bias, the result is that *t*_(47)_ = 1.39, *p* = 0.17, which means there is no significant bias. Our current meta-analysis reported a significant *p*-value based on 25 studies. According to Rosenthal's suggestion, we should compute how many missing studies we would need to retrieve and incorporate in the analysis before the *p*-value became non-significant, the number of the missing studies was called Rosenthal Fail-safe *N*. The larger number of studies that are needed to nullify the effect, the more confident we can be of a real effect (Rosenthal, 1979). The result of Fail-safe *N* showed that at least 973 studies with the opposite conclusions would be required to overturn the findings of this meta-analysis.

### Subgroup Analysis

Given the moderate heterogeneity of effect sizes and influence factors aforementioned in the introduction, we conducted subgroup analyses to examine whether the effect sizes varied according to measures of observers' self-attractiveness, type of stimulus, tasks of preference and same/opposite-sex stimuli. The present meta-analysis included only one effect size of same-sex feminine preferences in male stimuli and none of masculine preferences in female stimuli. Thus, we conducted subgroup analysis on feminine preferences for female stimuli and masculine preferences for male stimuli for the same-opposite sex analysis. Moreover, in masculine preference, all the effect sizes on relationship context and contraceptive use were extracted from the preference of opposite-sex (female preference for male stimulus); in feminine preference, all the data on relationship context also came from the preference of opposite-sex (male preference for female stimulus). The results of the subgroup analysis have been presented in Table 2. The moderated analysis showed that, for masculine (*x* = 39), task of preference, measures of observers' self-attractiveness, type of stimulus affected the association between observers' self-attractiveness and their masculine preferences, additionally, women's preference for male masculinity varied across contraceptive use/no and short-long term relationship context. And for feminine preference (*x* = 16), this association also depended on the task of preference, relationship context, same/opposite-sex stimuli.

**Table 2:** Summary of the results of the sub-group meta-analysis.

**Outcome**	**Moderator**	**Subgroup**	***X***			**Effect size (95% confidence interval)**			***Z***	***Q***	***I*^2^*(%)***	***p***
**Variable**						**Effect size(*r*)**	**lower**	**upper**				
Masculinity	MOOA											0.000
		Objective		13		0.133	0.055	0.210	3.325^***^	28.868	58.431	
		Subjective		26		0.069	0.044	0.092	5.578^***^	41.651	39.977	
	Task											0.000
		Attractive rating		9		0.048	−0.061	0.157	0.864	19.591	59.165	
		FCT and AR		7		0.145	0.036	0.250	2.610^**^	7.438	19.332	
		FCT		12		0.081	0.033	0.129	3.281^**^	21.468	48.761	
		ST		11		0.183	0.065	0.297	3.018^**^	16.686	40.070	
	Stimulus											0.000
		Body		2		0.185	0.044	0.319	2.568^**^	0.126	0	
		Face		29		0.078	0.032	0.123	3.321^***^	54.769	48.877	
		Voice		8		0.184	0.079	0.285	3.407^***^	9.499	26.309	
	Same/opposite gender	Male stimulus										0.000 (same/opposite sex)
		Same	3			0.066	0.008	0.122	2.246	1.827	0.00	
		Opposite	35	Effect of opposite		0.107	0.059	0.155	4.302	71.000	52.112	
				RC								0.028(RC)
				Short	9	0.001	−0.011	0.112	0.02	13.53	40.88	
				Long	9	0.228	0.098	0.350	3.41^***^	16.51	51.54	
				Contraceptive using								0.000 (Contraceptive using)
				No	10	0.183	0.100	0.263	4.295	10.455^**^	13.91	
				Yes	2	0.093	−0.232	0.399	0.552	3.26^***^	68.39	
Femininity	Task											0.000
		FCT and AR		4		−0.026	−0.238	0.188	−0.235	16.60	81.932	
		FCT		6		0.076	−0.039	0.188	1.300	42.069	88.115	
		ST		6		0.125	0.063	0.187	3.91^***^	5.835	14.315	
	Same/opposite gender	Female stimulus										0.005 (same/opposite sex)
		same	3			0.219	0.022	0.400	2.171^*^	17.6674^**^	88.676	
		opposite	7	Effect of opposite		0.083	0.008	0.156	2.179^*^	15.006^***^	60.016	
				RC								0.000 (RC)
				short	2	0.202	0.002	0.159	1.997^*^	0.93	0	
				long	3	0.081	0.084	0.314	3.327^**^	1.287	22.27	

x, number of effect sizes; MOOA, Measures of Observers' Own Attractiveness; RC, Relationship Context; FCT and AR, Forced-choice Test and Attractiveness Rating; FCT, Forced-choice Test; ST, Sequence Test;

*** *p < 0.001*,

** *p < 0.01*,

* *p < 0.05*.

### Meta-Regression Analysis

To assess the influence of observers' age, published year, and sample size of observers on the effect size (*r* coefficient), considering these three variables are continuous, meta-regression analysis were carried out for masculine preference and feminine preferences separately. Firstly, we looked the effect size of relationship between self-attractiveness and masculine preference as dependent variable. The results showed the effect size decreased with observers' age, published year, and sample size of observers (see Table 3). Specifically, the older observers were, the more negative relationship between self-attractiveness and masculinity preference. In other words, in younger people, individuals in high physical condition would more prefer masculinity. Further, the larger the sample size of the study, the weaker was this relationship, and the later the publishing year of the study, the smaller was the effect. Subsequently, the effect size of relationship between self-attractiveness and feminine preference was used as dependent variable. Only the publishing year significantly positively influenced the relationship.

**Table 3:** The results of the meta-regression analysis.

**Outcome variable**	**Moderator**	***x***	***B***	***P***
Masculinity preference	Observer's age	30	−0.00467	0.016
	Published year	39	−0.01185	0.000
	Sample size of observers	39	−0.00004	0.009
Femininity preference	Observer's age	16	0.00222	0.163
	Published year	16	0.02641	0.004
	Sample size of observers	16	0.0004	0.402

## Discussion

### The Overall Association

Human sexual dimorphic preference is condition-dependent, and it is an attractive-contingent preference. However, the empirical evidence is inconsistent. The present meta-analysis provides a quantitative synthesis of the available evidences on the attractive-contingent preference, and reveals a significant overall relationship (*r* = 0.095, *x* = 55). Additionally, self-attractiveness is significantly positively but weakly related with masculine and feminine preference (masculinity: *r* = 0.102, *x* = 39; femininity, *r* = 0.076, *x* = 16). These findings are consistent with the concept of condition-dependent preference. Condition dependence lies at the heart of the trade-off between costly sexual traits and other major fitness components such as survival and growth. Variability among individuals' physical condition can potentially influence the form, direction, and intensity of sexual selection in the population as a whole (Widemo and Sæther, 1999). Some previous studies have confirmed that high-quality females were more attracted to markers of quality in males (masculine men) across different domains (face, voice, body, and smell) (Little et al., 2011b), which is due to the fact that their own high attractiveness means that lower parental investment is less detrimental. Actually, there is a more complex relationship between self-condition and mating choice, as self-attractiveness does not occur in isolation (Little et al., 2014). The objective or subjective measurement of self-attractiveness (Smith et al., 2009b), relationship context of preference for opposite-sex stimuli (Little et al., 2007; Kandrik and DeBruine, 2012), type of stimuli would also play an important role in observers' sexual dimorphic preferences.

### The influence of Same/Opposite-Sex, Relationship Context, Contraceptive Use: Basing on the Life History Tradeoff Strategies

As shown in Table 2, the intensity of attractive-contingent preference did change across same/opposite-sex conditions, which is inconsistent with our hypothesis. In masculine preference for male stimulus, the correlation between women's self-attraction and male masculine preference was stronger than that of men's self-attraction (opposite = 0.107, *x* = 35; same = 0.066, *x* = 3). Patterns of women preferences for men's masculine features are more complex (Burke and Sulikowski, 2010; Holzleitner and Perrett, 2017). On the viewing of LH strategies, good genes, and good provisioning male mate attributes evolved mainly from polygyny: muscularity in men indicating higher immune competence, physical attractiveness, and dominance, competition, high status, but they attract and tend to have more female mates at the same time, and they invest less in each female. In spite of this, attractive women are more confident and believe that they can offset the costs of choosing good genes and good providing partner (Little et al., 2001). Although, attractive men more likely to prefer masculine men as social allies, self-rated sex typicality is the stronger trait to predict preference for sex-typical physical cue in same-sex faces (Kandrik and DeBruine, 2012), and appears to be more important influence factor of men's preference for same-sex physical cues. Moreover, the market value (good physical condition) is potentially a more important resource for women's mating choice than for men's cooperative choice (Kandrik, 2017). Otherwise, When it comes to the preference for female femininity, the influence of same-sex is greater than that of opposite-sex (same = 0.219, *x* = 3; opposite = 0.083, *x* = 7), in another word, attractive women showed a more obvious trend than men did. Possibly because more attractive women may perceive less threat from other feminine women and more likely to look them as social allies (Fisher, 2004). While Attractive men tend to choose charming women but also trade off the benefit and the cost of cuckoldry. Therefore, men's choices also depend on the context of the relationship (long or short term) (Burriss et al., 2011; Little et al., 2014).

In line with our expectations, on women's preferences in male masculinity, long-term context had a larger effect size as compared to the short-term context (correlation in the short-term context was 0.001, *x* = 9; that in the long-term context was 0.228, *x* = 9). Attractive women expressed preferences for all three clusters of men's mate characteristic (3Gs) (Buss and Shackelford, 2008), as the aforementioned conflict of good genes, good provider and good father, they have to trade off. Women placed more value on man's physical attractiveness, muscularity and immediate resource displays (Haselton and Gangestad, 2006) when pursuing short-term mating, in contrast, they placed greater importance on resource acquisition potential and good dad indicators when pursuing long-term mating (Buss and Shackelford, 2008; Lu et al., 2015). In the context of short-term sexual relationships, the perceived cues to high parental investment in feminine men are of little value to women. Moreover, women of higher/lower physical condition can extract potential benefits from masculine men by copulating and conceiving with a short-term relationship. And therefore, both attractive and unattractive females trade on good genes (masculinity) in short-term context. On the contrary, in long-term relationships, better parenting and increased cooperation may outweigh the benefits of genetic fitness, thereby enhancing the attractiveness of more feminine males (Little and Mannion, 2006). Highly attractive women think that they are good enough to find another partner soon, and therefore, they do not need to be restricted by the real conditions or change their preferences according to the relationship context. However, women with poorer appearance have to trade off good genes and parental investment in the long-term context. As a result, the attractive-contingent masculine preference appeared more apparent in the long- than in the short-term context. A similar trend was observed in the voice domain (Feinberg et al., 2012).

On men's preference for women's femininity, the effect size for the short-term context was larger as compared to that for the long-term context (short-term = 0.202, *x* = 2; long-term = 0.081, *x* = 3), which indicated that attractive men were more strongly attracted by feminine female in short-term relationship than unattractive men, however, in long-term context, the men's self-attractiveness wasn't closely associated to their preference for femininity. Firstly, on the perspective of LH tradeoff strategies, preferences represent various LH strategies: fertility-related attributes (good genes) represent a fast LH strategy, whereas attributes of good parenting serve as slow LH function (Lu et al., 2017). Attractive men tended to adopt fast LH strategies with feminine women in short-term relationship, reported more short-term partners than less attractive men (Rhodes et al., 2005). As without regards to raising the young, their attractiveness primed them with chance to produce greater number of offspring. Secondly, unlike women trading off between man's good gens and his resource, willing to invest, a man risked raising a child which is not his own, they mainly traded on partner's physical attractiveness and personality traits in long-term relationship (Little et al., 2014). Because feminine women are perceived as unfaithful and are considered more likely to have an affair or a brief sex trade. Both attractive and unattractive men considered women's risk of cuckoldry carefully in long-term contexts, such that self-attractiveness seemed less related with femininity preferences (Little et al., 2014).

The analysis of the moderating effect showed that the correlation coefficient for the relationship of the self-reported attractiveness of non-users of contraceptives and their masculinity preference was significantly higher than that of users (0.183 vs. 0.093). A cross-sectional longitudinal study confirmed that hormonal contraceptive users had a weaker preference for masculinity than non-users did (Little et al., 2013), which was consistent with the findings of other previous studies (Roberts et al., 2014). In addition, hormonal contraceptives tended to decrease women's overall physical attractiveness (Puts and Pope, 2013; Welling, 2013; Roberts et al., 2014), and as discussed above, women's attractiveness was their “market value” and an important factor in “intra-sexual competition.” Therefore, by diminishing women's attractiveness, hormonal contraceptives might make it more difficult for women to compete for romantic partners (Smith et al., 2009a; Puts and Pope, 2013). Similarly, effect size was more negatively correlated with observers' age in masculine preference (see Table 3). The observers in the present analysis pool for masculine preference were females. In other words, younger attractive women preferred masculine faces. As people age, especially women, they recalibrate subjective impressions of their own attractiveness (i.e., impressions of their own “market value”), which, in turn, leads to a recalibration of their mate preferences (Little et al., 2011a). With a decrease in their market value with age, most women pay attention to parental characteristics (Jones et al., 2011).

### Type of Stimuli

With reference to feminine preferences, because the present meta-analysis included only one study on vocal and none on body stimuli, we examined the moderating role of stimuli type in masculine preference. This analysis revealed significantly different effect sizes for body, voice, and face stimuli. Specifically, the effect sizes for voice and body were larger than that for face stimuli (body = 0.185, *x* = 2; voice = 0.184, *x* = 8; face = 0.078, *x* = 29), the effect sizes of body and voice are close to 0.2, a moderate amount. However, the coefficient of face and masculine preference was 0.078, a weak correlation, which was out of our expectation. Previous studies have interpreted covariation as evidence that different domains of masculinity all advertised a common underlying factor. Additionally, we suggested that men's and women's masculinity, as signaled by multiple traits, were related to some common information about the underlying quality of the observed individual. Of course, this did not mean that the signals overlap perfectly (Little et al., 2011a), and indeed, our data suggests that masculine preference in all the three traits was strongly related to observers' self-attractiveness. This indicates a distinct characteristic. Masculine facial preferences are less relevant to self-attractiveness, potentially because a large number of studies have focused on facial masculine preference, while little attention has been paid to the preference for masculinity in the voice and body of the observed. Furthermore, most of these studies on facial preferences regarded self-attractiveness as a covariant, and they explored its interaction with other main variables (e. g., relationship context or menstrual cycle). Evidently, the correlation varied in different conditions, and therefore, more effect sizes of facial masculine preference (*x* = 29) averaged into a smaller effect size.

### The Task of Preference and Measures of Self-Attractiveness

Stronger effects were found when the sequence test (ST) was used instead of using the forced-choice test (FCT) with an attractiveness rating (Masculinity: ST = 0.183 *x* = 11; FCT = 0.081, *x* = 12; Femininity: ST = 0.125 *x* = 6; FCT = 0.076, *x* = 6). The sequence test provides observers a program in which they can regulate the sexual dimorphism independently until they reached the level that they considered most attractive (Carrito et al., 2016). The forced-choice test with an attractiveness rating merely provides observer two dimensions of a face (masculine and feminine) to choose from. They must then choose one that they consider more attractive and rate its attractiveness (Little and Mannion, 2006). Thus, the sequence test is more ecological and it reflects the observer's preference for dimorphism more clearly.

Most studies included in the present meta-analysis focused on masculine preferences and none used an objective index to measure feminine preferences. Thus, we conducted this subgroup analysis only on masculine outcomes. A moderator analysis revealed a significant difference in the effect sizes of masculine preferences according to the measures of self-attractiveness, which is consistent with Penton-Voak et al.'s findings (Penton-Voak et al., 2003). They thought objective measures could be independent from menstrual cycle, and would be more stable than self-assessments are (Penton-Voak et al., 2003). Some researchers have emphasized that BMI and WHR were better indicators of female attractiveness (Swami et al., 2007; Smith et al., 2009b). Similarly, in the current meta-analysis, objective attractiveness was highly related to masculine preferences (effect size = 0.133, *x* = 13), but self-rated attractiveness showed a weak relationship (effect size = 0.069, *x* = 26). Most of the participants were female in the studies included in this meta-analysis. Subjective measures may be influenced by their individual physiology, for example, women perceived themselves more attractive when ovulating (Singh et al., 2001), and self-rated attractiveness may potentially fluctuate in a short space of time. Self-report measures are subjective and may not reflect how an individual is perceived by others. Therefore, objective measurements such as WHR, BMI, and other-rated attractiveness may be more sensitive to the relationship between self-attractiveness and sexual dimorphic preferences.

## Limitations

We would like to mention some limitations of the present meta-analysis. The first concerns the number of studies analyzed. We included 25 studies, which were divided into sets based on feminine and masculine preferences before submitting them to separate meta-analyses. This meant that we had a limited number of studies for each moderator level (see Table 2). Therefore, some levels of the moderator were under-represented. The second limitation of the current meta-analysis is that the age range of observers was relatively concentrated, in that most of them were teenagers or young individuals in their 20s, and only two studies selected older subjects with an average age of 33 years (Zietsch et al., 2015) and 48 years (Jones et al., 2011). Therefore, although the results of the present study revealed a significant regulatory effect of age, the accuracy of this correlation is difficult to prove.

## Conclusions

This meta-analysis suggests that there is a real relationship between sexual dimorphic preferences and observers' self-attractiveness. Therefore, in future studies, researchers should control self-attractiveness to ensure the reliability of experimental results. According to the results of the present subgroup analyses, this relationship depends on relationship context, same/opposite-sex, and contraceptive using. These three moderating effects represented the observer's trade-off on 3Gs, were consistent with the life history strategies. Besides, measurement of observers' attractiveness, type of preference task and stimuli may also may involve the relationship. Therefore, in future studies, researchers should consider these factors and their interactions.

## Author Contributions

LC and ZR co-designed the study and wrote the manuscript. LC and YY conducted the literature searches, the studies selection. LC and XJ took charged. LC, XJ, and HF analyzed the data. XJ and LC revised according the review.

### Conflict of Interest Statement

The authors declare that the research was conducted in the absence of any commercial or financial relationships that could be construed as a potential conflict of interest.
