# Supplementary material for: The Relationship Between Observers' Self-Attractiveness and Preference for Physical Dimorphism: A Meta-Analysis
Source: Front Psychol. 2018 Dec 5;9:2431. doi: 10.3389/fpsyg.2018.02431 (PMC6290584; doi:10.3389/fpsyg.2018.02431)
Supplement: Supplementary file 2 [file Table_2.docx]

Supplementary Material

**The relationship between observers’ self-attractiveness and preference for physical dimorphism: A meta-analysis**

Lijun Chen*^1, 2^ Ying Yang^1^, Huiyong Fan^3^, Xiaoliu Jiang^1^, Zhihong Ren^4,5*^

*** Correspondence:** Zhihong Ren

psyren@qq.com

# Supplementary Data

All datasets for this study are included in the manuscript and the supplementary files.

The data used in the manuscript is stored in the [self-attractiveness and dimorphic preference.xls] file. If required, please download it from the Supplementary material. The tables and Figures presented in the manuscript are all analyzed from the data set. We used the CMA software to analyze.

# Supplementary Figures





**Supplementary Figure 1.** **Forest plot of the studies of masculine preferences(*x* = 39)**.





**Supplementary Figure 2.** **Forest plot of the studies of feminine preferences(*x* = 16)**.


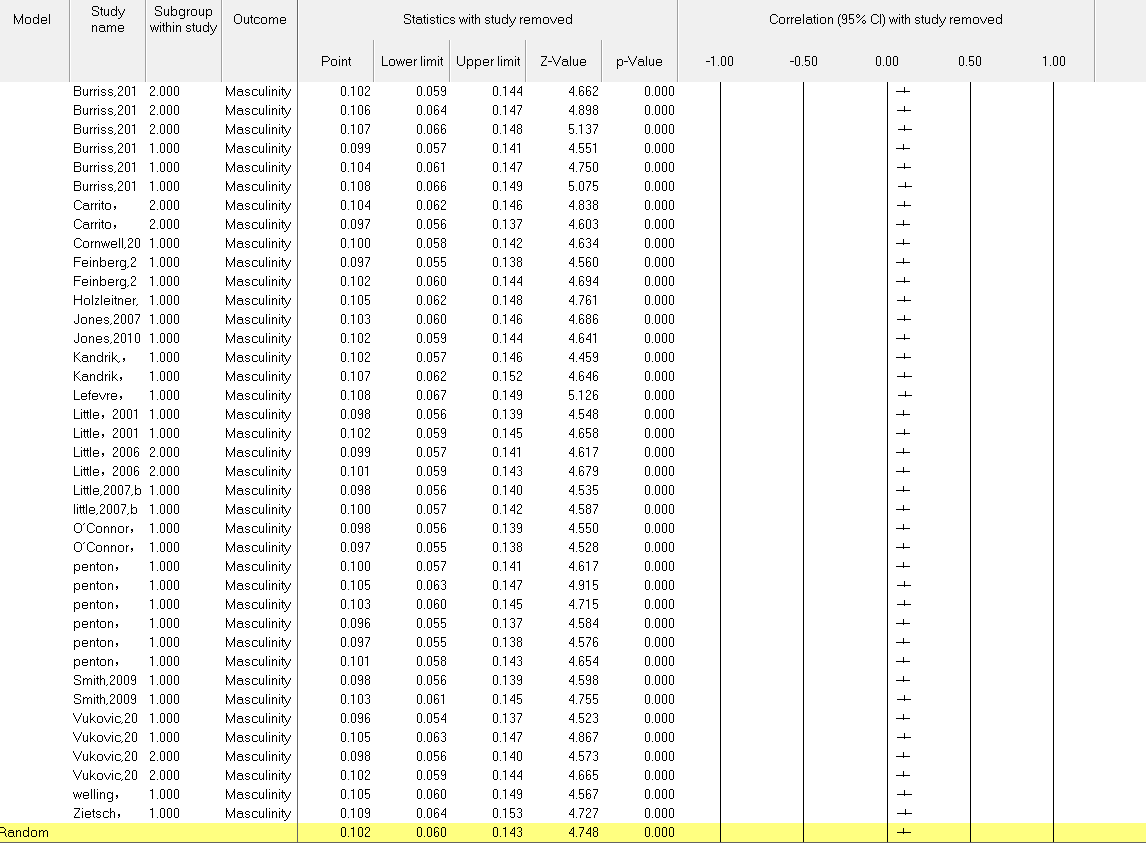


**Supplementary Figure 3.** **Sensitivity analysis for the studies of masculine preferences(*x* = 39)**


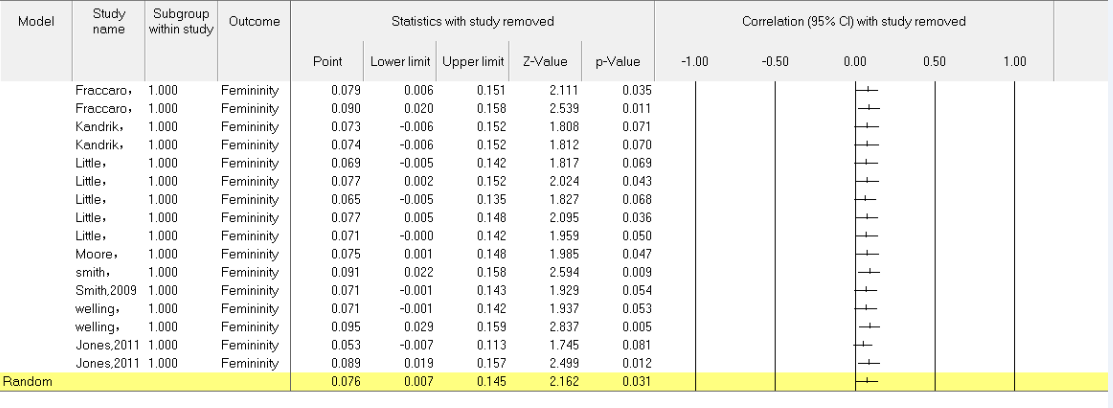


**Supplementary Figure 4.** **Sensitivity analysis for the studies of feminine preferences(*x* = 16)**.
